# Role of Serum E-Selectin as a Biomarker of Infection Severity in Coronavirus Disease 2019

**DOI:** 10.3390/jcm10174018

**Published:** 2021-09-06

**Authors:** Alessandra Oliva, Emanuele Rando, Dania Al Ismail, Massimiliano De Angelis, Francesca Cancelli, Maria Claudia Miele, Raissa Aronica, Vera Mauro, Federica Di Timoteo, Lorenzo Loffredo, Claudio M. Mastroianni

**Affiliations:** 1 Department of Public Health and Infectious Diseases, Sapienza University of Rome, Piazzale Aldo Moro 5, 00185 Rome, Italy; rando.1634464@studenti.uniroma1.it (E.R.); dania.alismail@uniroma1.it (D.A.I.); massimiliano.deangelis@uniroma1.it (M.D.A.); francesca.cancelli@uniroma1.it (F.C.); mariaclaudia.miele@uniroma1.it (M.C.M.); raissa.aronica@uniroma1.it (R.A.); vera.mauro@uniroma1.it (V.M.); federica.ditimoteo@uniroma1.it (F.D.T.); claudio.mastroianni@uniroma1.it (C.M.M.); 2 Sapienza School for Advanced Studies (SSAS), Sapienza University of Rome, Viale Regina Elena, 291, 00161 Rome, Italy; 3 Department of Clinical, Internal Medicine, Anaesthesiology and Cardiovascular Sciences, Sapienza University of Rome, 00185 Rome, Italy; lorenzo.loffredo@uniroma1.it

**Keywords:** SARS-CoV-2, E-selectin, COVID-19, endothelial activation

## Abstract

Introduction: E-selectin is a recognized marker of endothelial activation; however, its place in Coronavirus Disease 2019 (COVID-19) has not been fully explored. Aims of the study are to compare sE-selectin values among the Intensive Care Unit (ICU)-admitted and non-admitted, survived and non-survived patients and those with or without thrombosis. Methods: A single-center study of patients with COVID-19 hospitalized at Policlinico Umberto I (Rome) from March to May 2020 was performed. Simple and multiple logistic regression models were developed. Results: One hundred patients were included, with a median age (IQR) of 65 years (58–78). Twenty-nine (29%) were admitted to ICU, twenty-eight (28%) died and nineteen (19%) had a thrombotic event. The median value (IQR) of sE-selectin was 26.1 ng/mL (18.1–35). sE-selectin values did not differ between deceased and survivors (*p* = 0.06) and among patients with or without a thrombotic event (*p* = 0.22). Compared with patients who did not receive ICU treatments, patients requiring ICU care had higher levels of sE-selectin (36.6 vs. 24.1 ng/mL; *p* < 0.001). In the multiple logistic regression model, sE-selectin levels > 33 ng/mL, PaO_2_/FiO_2_ < 200 and PaO_2_/FiO_2_ 200–300 were significantly associated with an increased risk of ICU admission. sE-selectin values significantly correlated with a neutrophil count (R = 0.32 (*p* = 0.001)) and the number of days from the symptoms onset to hospitalization (R = 0.28 (*p* = 0.004)). Conclusions: sE-selectin levels are predictive of ICU admission in COVID-19 patients. Since data on the relation between sE-selectin and COVID-19 are scarce, this study aims to contribute toward the comprehension of the pathogenic aspects of COVID-19 disease, giving a possible clinical marker able to predict its severity.

## 1. Introduction

On December 2019, a novel coronavirus, named severe acute respiratory syndrome coronavirus 2 (SARS-CoV-2), was identified as a cause of viral pneumonia in the city of Wuhan, China [1]. Since then, the resulting Coronavirus Disease 2019 (COVID-19) has been responsible for a pandemic with a high global burden in terms of morbidity and mortality. COVID-19 can result in a spectrum of forms ranging from a respiratory tract infection to a systemic and severe form characterized by acute respiratory distress syndrome, shock and thrombotic events [2,3,4]. A great number of these phenomena have been investigated and considerable attention has been paid to the vascular manifestations of the disease [5,6]. Multiple studies have illustrated SARS-CoV-2 to target epithelial cells causing local inflammation leading to an imbalance between anti-coagulant and pro-thrombotic factors [7]. E-selectin is a recognized marker of endothelial activation [8] which has been studied in cardiovascular disease [9], but its place in COVID-19 has not completely understood.

Based on these premises, we aim to (i) evaluate the levels of serum E-selectin (sE-selectin) in COVID-19-hospitalized patients as marker of endothelial activation and (ii) to correlate the values of this biomarker with the severity of COVID-19, the in-hospital mortality rate and the occurrence of thrombotic events, adding new evidences to the comprehension of some aspects of SARS-CoV-2 infection pathogenesis. 

## 2. Materials and Methods

### 2.1. Study Population

Over a 2-month period (March–May 2020), patients with COVID-19 hospitalized at the Azienda Policlinico Umberto I, Sapienza University of Rome, were enrolled in this study. Demographic, clinical and radiological data from all participants were anonymously recorded in an electronic database. Patients were further divided according to disease severity (defined as ICU admission), in-hospital mortality and the occurrence of thrombotic events during hospitalization. 

### 2.2. Definitions

Diagnosis of COVID-19 was determined based on suggestive clinical symptoms plus detection of SARS-CoV-2 RNA in nasopharyngeal swab samples by using real-time RT-PCR assay (RealStar SARS-CoV-2 RT-PCR, Altona Diagnostics GmbH, Mörkenstr.12, 22767 Hamburg, Germany). All tests and procedures were performed following the manufacturers’ protocols. Disease severity was defined as ICU admission over hospitalization and included need for orotracheal intubation, severe respiratory distress or shock. Arterial and/or venous thrombotic/embolic events included, among others, pulmonary thrombo-embolism, myocardial infarction, acute brain ischemia and acute limb ischemia. Thrombotic/embolic events were defined as the appearance of new ischemic/embolic events diagnosed as follows: (1) pulmonary thrombo-embolism by lung computed tomography (CT) scan [10]; (2) myocardial infarction by EKG changes associated with enhanced markers of cell necrosis [11]; (3) acute brain ischemia by onset of new focal neurological signs and symptoms and confirmed, whenever possible, by magnetic resonance or CT imaging [12]; (4) acute limb ischemia [13]. 

### 2.3. Marker of Endothelial Activation: sE-Selectin

For each subject, whole blood samples were collected at hospital admission (T0). Ten milliliters of whole blood were collected by venipuncture in Vacutainer tubes containing ethylene-diamine-tetra-acetic acid (EDTA) (BD Biosciences, San Jose, CA, USA) at each study visit. Plasma was immediately separated by centrifugation at 2000 rpm for 10 min and further stored at −80 °C until the assays were performed. Serum E-selectin levels were evaluated using enzyme-linked immunosorbent assays in plasma, according to manufacturers’ instructions. Values of sE-selectin were expressed as ng/mL (sample diluted 1:10). The study protocol was approved by the local Ethics Committee (ID Prot. 298/2020). 

### 2.4. Statistical Analysis

The primary analysis aimed to investigate clinical and laboratory characteristics in two groups defined as ICU admission vs. non-ICU admission. Continuous variables were described using median and interquartile ranges, categorical variables using frequencies and percentages. Wilcoxon rank sum test was used to compare continuous variables and χ2 test for categorical variables. A *p*-value of < 0.05 was used to consider differences statistically significant.

Secondary analysis was performed to ascertain risk factors associated with ICU admission. Since imputation was not feasible as a result of the restricted sample, variables having more than eight missing values were excluded from the regression analysis to guarantee an appropriate number of observations. To evaluate ICU admission prediction performance of sE-selectin levels, a simple logistic regression model was developed and odds ratio, 95% confidence intervals and receiver operating characteristic (ROC) curves with area under the curve (AUC) were measured. Furthermore, to determine an appropriate cutoff value, we used Youden’s Index, grouping sE-selectin levels by two.

We performed a multiple logistic regression model using only statistically significant variables identified with single logistic regression analyses. Finally, PaO_2_/FiO_2_ grouped by two and a value of sE-selectin greater than 33 ng/mL were selected for the multiple regression model using a stepwise selection procedure. Odds ratios and 95% confidence intervals (CI) were calculated, although CI were not reliable since an automated variable selection procedure was used. To assess the model’s discriminative ability, accuracy, ROC curve and AUC were measured.

Statistical analyses were performed with R software version 4.0.0 and RStudio version 1.3.95 (R Core Team (2020). R: A language and environment for statistical computing. R Foundation for Statistical Computing, Vienna, Austria. Available online: https://www.R-project.org/ (accessed on 16 May 2021)).

## 3. Results

### 3.1. Patients’ Characteristics

**Figure 1:**
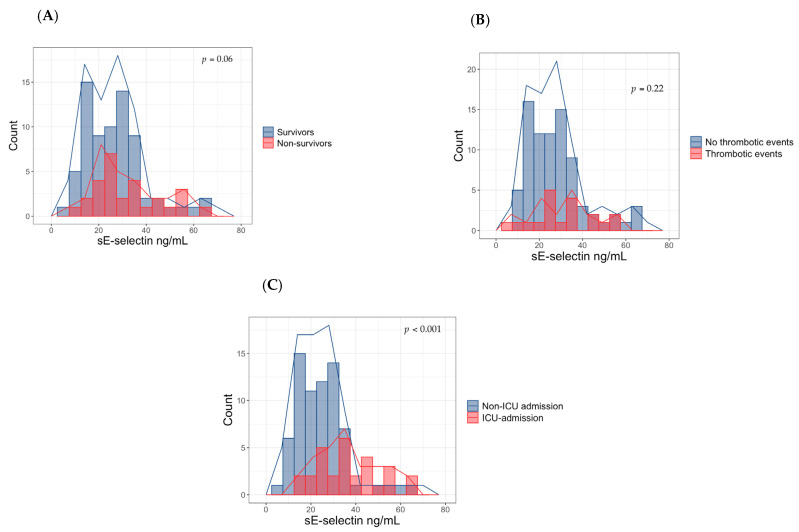
Serum E-selectin levels in non-survivors and survivors (**A**), in patients with thrombotic events and those without thrombotic events (**B**) and in ICU and non-ICU-admitted patients (**C**). ICU: intensive care unit.

A total of 100 patients with a COVID-19 diagnosis were included in our study. The median age (IQR) was 65 (58–78) years, 62 (62%) were men. Of these, 29 (29%) were admitted in ICU, 28 (28%) died and 19 (19%) had a thrombotic event. Thrombotic/embolic events included a pulmonary thrombo-embolism (*n* = 5), myocardial infarction (*n* = 3), acute brain ischemia (*n* = 2), acute limb ischemia (*n* = 7) or other (*n* = 2, thrombophlebitis, *n* = 1 and gonadal vein thrombosis, *n* = 1). The median value (IQR) of sE-selectin was 26.1 ng/mL (18.1–35). Median sE-selectin values did not differ between the deceased and survivors (27.1 (22.4–41.7) vs. 25.2 (17.1–32.6) ng/mL, *p* = 0.06) and among patients who experienced a thrombotic event and who did not (32.1 (23.5–39.9) vs. 26.1 (17.5–34.4) ng/mL, *p* = 0.22) (Figure 1A,B). Other baseline characteristics are reported in Table 1.

### 3.2. Comparison between ICU and Non-ICU-Admitted Patients 

The between-group differences among ICU-admitted and non-ICU-admitted patients are shown in Table 1. No differences in age, lymphocytes, monocytes and platelets count, fibrinogen, comorbidities prevalence, Charlson Comorbidity Index and use of ACE inhibitors, angiotensin receptors blockers (ARBs), diuretics, antiaggregants, anticoagulants and statins were found to be statistically significant. On the other hand, sE-selectin levels, male sex, the peripheral saturation of oxygen, PaO_2_/FiO_2_ ratio, white blood cells count, polymorphonuclear neutrophils count, serum creatinine values, albumin, lactate dehydrogenase (LDH) and D-dimer levels were significantly different between the two groups. Compared with patients who did not receive intensive care treatments, patients requiring ICU care had higher levels of sE-selectin (36.6 (25.8–47.1) vs. 24.1 (17.0–30.1) ng/mL; *p* < 0.001) (Figure 1C). 

### 3.3. sE-Selectin for Prediction of ICU Admission

A simple logistic regression model with sE-selectin levels as the predictor of ICU admission was developed. Each increment of 1 ng/mL of sE-selectin level was associated with a higher risk of ICU care with an odds ratio of 1.07 (95% CI, 1.03 to 1.12) (Table 2). The AUC of the ROC curve was 0.75 (95% CI, 0.64 to 0.87). The ROC curve for the differentiation of ICU-admitted from non-admitted patients, the respective AUC and test performance of sE-selectin for prediction are shown in Table 2 and Table 3, respectively.

### 3.4. Multiple Logistic Regression Analysis

**Figure 2:**
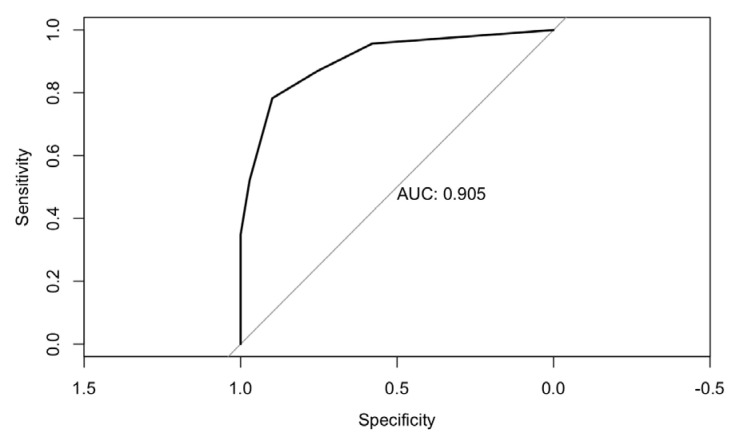
ROC curve of multiple logistic regression model using sE-selectin > 33 ng/mL, PaO_2_/FiO_2_ 200–300 and PaO_2_/FiO_2_ < 200 as predictors of Intensive Care Unit (ICU) admission.

A multiple logistic regression model using a stepwise selection procedure was calculated. Odds ratios and 95% confidence intervals of both univariate and multivariate models are reported in Table 4. A value of sE-selectin levels greater than 33 ng/mL (OR 13.7 (95% CI, 3.2 to 81.9)), a PaO_2_/FiO_2_ ratio below 200 (OR 80.3 (95% CI, 14.4 to 716.2)) and a PaO_2_/FiO_2_ ratio between 200 and 300 (OR 9.1 (95% CI, 1.7 to 62.1)) were found to be significantly associated with an increased risk of ICU admission, after being adjusted for sex, LDH levels and the absolute neutrophil count. The resulting AUC of the ROC curve was 0.90 (95% CI, 0.83 to 0.98) (Figure 2).

### 3.5. Correlation Analyses

**Figure 3:**
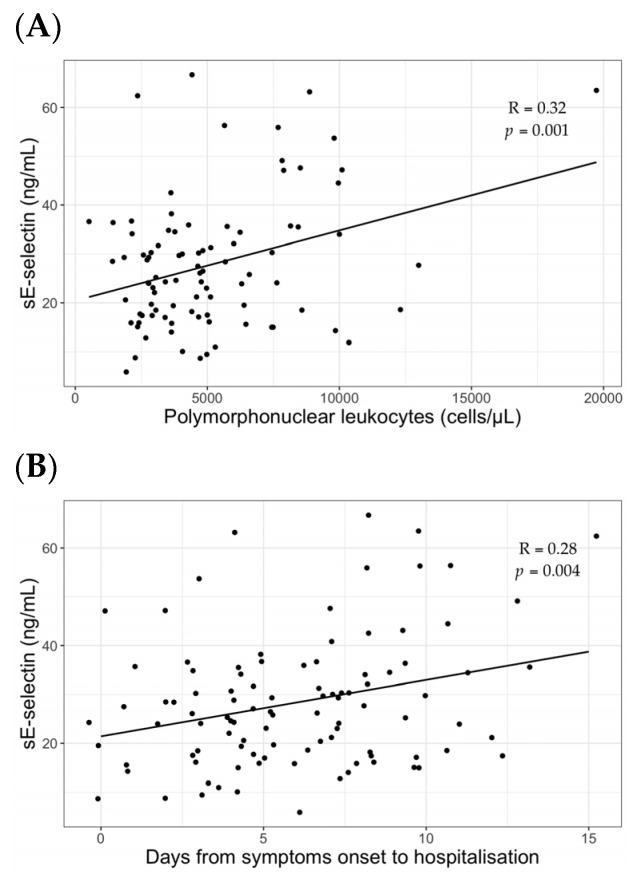
Correlation analyses of sE-selectin with polymorphonuclear leukocytes count (**A**) and days from symptoms onset to hospitalization (**B**).

Correlation coefficients of variables included in the multiple regression model were calculated. Values of sE-selectin significantly correlated with the neutrophils count with a coefficient of 0.32 (*p* = 0.001) (Figure 3A). In addition, a positive correlation between sE-selectin values and the number of days from the symptoms onset to hospitalization was found with a correlation coefficient of 0.28 (*p* = 0.004) (Figure 3B). Although not significantly, sE-selectin positively correlated with D-dimer levels (coefficient 0.17, *p* = 0.08).

## 4. Discussion

This study showed that sE-selectin levels are predictive of ICU admission in COVID-19 patients, providing a possible biomarker to determine the severity of the disease. Indeed, compared to patients who did not require intensive care treatment, the results demonstrated higher levels of sE-selectin in ICU-admitted patients. The sE-selectin (Endothelial Leukocytes Adhesion Molecule (1) is a soluble adhesion molecule synthetized by activated endothelium, known to have pro-thrombotic effects involved in coagulopathy [9,14]. Thrombotic complications are frequent during the course of the disease contributing to its morbidity and mortality [15,16] and several mechanisms have been studied as a cause of these phenomena [17,18,19,20]. Vascular inflammation and endothelial activation have been related to the spectrum of COVID-19-associated coagulopathy [5]. Furthermore, among others, the role of sE-selectin as a marker of endothelial dysfunction, inflammation or damage, points out the central role of the endothelium itself not only in the development of a more severe form of the disease, but also in the pathogenesis of the cardiovascular events that occur during COVID-19, including immuno-thrombosis and arrhythmias [6]. As a matter of fact, the inflammatory milieu as a consequence of the so-called ‘cytokine storm’ may contribute to the progression of endothelial damage, which, in turn, amplifies the pro-inflammatory and pro-coagulant processes [21] as well as the high production of reactive oxygen species [18], all conditions observed during severe COVID-19. 

Among patients admitted in the intensive care unit sE-selectin levels were significantly higher. This finding was not surprising since endothelial activation may reflect a more severe form of the disease, possibly leading to ICU admission. So far, only a few studies investigated the prognostic effect of sE-selectin as a marker of disease severity or mortality in COVID-19 patients [21,22,23], although with non-univocal results [24]. In fact, similarly to our results, Smadja and colleagues showed that sE-selectin at hospital admission was a discriminant biomarker for ICU admission [22]; likewise, Vassiliou et al. found that, amongst several markers of endothelial damage, sE-selectin was significantly elevated in ICU non-survivors compared to survivors, possibly predicting mortality in critically ill COVID-19 patients [23]. More recently, Birnhuber et al. showed significantly higher levels of sE-selectin in critically ill COVID-19 patients than healthy controls [21]. On the other hand, Spadaro and co-authors found that sE-selectin plasma levels did not differ between COVID-19 non-survivor and survivor patients with acute respiratory distress syndrome [24]. All in all, while the majority of the aforementioned studies converged with the concept that endothelial cell activation and dysregulation occur in COVID-19 and that markers of endothelial activation may have a prognostic role during the disease, still definite evidence is lacking. 

Hence, our results may provide additional insights into the pathogenesis of COVID-19 and its clinical implications.

It is also worth noticing that, although we did not find a significant difference between patients with and without thrombotic events, the median values tended to diverge (32.1 ng/mL vs. 26.1 ng/mL). The observed difference in sE-selectin values between subjects experiencing—or not—a thrombotic event may be a reflection of the pathophysiologic role of E-selectin in SARS-CoV-2 infection and may explain the high incidence of thrombosis in some patients in the clinical practice. Moreover, we found a positive correlation between neutrophils and sE-selectin values. The polymorphonuclear leukocyte activation in severe infections can lead to neutrophil extracellular traps (NET) formation [25]. NETs are web-like chromatin structures involved in the immune and anti-infective response [26,27]; their role in viral infections has already been investigated for Influenza and Respiratory syncytial virus (RSV) and consists of inhibiting viral replication and aggregation [28,29]. However, when NETs are excessively released, their activation might have detrimental effects to the host, being linked to endothelial cell injury [30,31,32]. Thus, our result could be explained by the detrimental effects of NETs on the endothelium in SARS-CoV-2 severe infection leading to its higher expression and participation in vascular damage, as illustrated in previous articles [33,34,35]. Finally, we showed that time from the symptoms onset to hospitalization significantly correlates with sE-selectin, highlighting a plausible relation between endothelium activation and disease progression. Nevertheless, our study should be interpreted considering several limitations. First, it was a retrospective study having the shortcomings of similar researches. Second, data were collected during the first months of the pandemic, possibly resulting in conclusions not completely reliable in the present phase, where the approach to COVID-19 is more solid and established. Third, the sample size was likely not large enough to catch important difference in outcomes, including death and thrombotic events, especially the latter, since sE-selectin distributions seemed to be different in the two groups despite not reaching a statistical significance. Fourth, we only dosed serum E-selectin at the beginning of the disease and not during its course. Indeed, we were not able to evaluate the possible influence of anticoagulation on sE-selectin levels and, consequently, to investigate in depth the relation between sE-selectin, the use of anticoagulants and thrombosis development. 

Except for the mentioned limitations, data on the relation between sE-selectin and COVID-19 as well as studies exploring the linkage between blood cells and the disease are scarce. As such, this study aimed to contribute toward the comprehension of pathogenic aspects of the illness, giving a possible clinical marker able to predict its severity. However, further studies are crucial to clarify the ample aspects of COVID-19 pathogenesis. 

## Figures and Tables

**Table 1:** Patient characteristics.

Characteristics	Overall, *N* = 100 ^1^	ICU Admission		*p*-Value ^2^
		Non-ICU ^1^	ICU ^1^	
Demographic				
Age (years), median (IQR)	65 (58, 78)	64 (58, 78)	68 (58, 75)	0.97
Male sex, *n* (%)	62/100 (62)	38/71 (54)	24/29 (83)	0.006
Comorbidities, *n* (%)				
Diabetes	18/96 (19)	13/71 (18)	5/25 (20)	0.85
CAD	13/96 (14)	9/71 (13)	4/25 (16)	0.74
ISH	42/96 (44)	31/71 (44)	11/25 (44)	0.98
CHF	12/96 (12)	6/71 (8.5)	6/25 (24)	0.073
AF	6/96 (6.2)	5/71 (7.0)	1/25 (4.0)	>0.99
PAD	16/96 (17)	12/71 (17)	4/25 (16)	>0.99
CVA/TIA	3/96 (3.1)	2/71 (2.8)	1/25 (4.0)	>0.99
Dementia	9/95 (9.5)	8/71 (11)	1/24 (4.2)	0.44
Asthma	7/96 (7.3)	6/71 (8.5)	1/25 (4.0)	0.67
COPD	14/92 (15)	11/70 (16)	3/22 (14)	>0.99
Liver disease	6/96 (6.2)	6/71 (8.5)	0/25 (0)	0.33
Hemiplegia	2/96 (2.1)	1/71 (1.4)	1/25 (4.0)	0.46
Solid tumor (last 5 years)	11/92 (12)	10/70 (14)	1/22 (4.5)	0.29
Leukemia/Lymphoma	6/96 (6.2)	5/71 (7.0)	1/25 (4.0)	>0.99
CCI	4 (1, 6)	4 (1, 6)	3 (2, 4)	0.25
Onset of symptoms to				
Hospitalization (days), median (IQR)	6 (4, 8)	5 (3, 8)	6 (4, 8)	0.67
Signs and symptoms, *n* (%)				
Cough	52/96 (54)	38/71 (54)	14/25 (56)	0.83
Dyspnea	48/97 (49)	30/71 (42)	18/26 (69)	0.019
Diarrhea	11/96 (11)	10/71 (14)	1/25 (4.0)	0.28
Fever	89/98 (91)	62/71 (87)	27/27 (100)	0.060
Fatigue	17/96 (18)	14/71 (20)	3/25 (12)	0.55
Blood gas analysis, median (IQR)				
SpO_2_ (%)	96 (92, 97)	97 (94, 98)	91 (85, 97)	<0.001
PaO_2_/FiO_2_ ratio	324 (242, 403)	352 (295, 424)	185 (117, 273)	<0.001
Laboratory, median (IQR)				
WBCs (μL)	5740 (4490, 8250)	5370 (4390, 6830)	8790 (4915, 10,685)	0.005
PMNLs (μL)	4590 (2910, 6390)	4050 (2780, 5122)	7560 (3768, 9840)	0.002
Lymphocytes (μL)	780 (560, 1140)	800 (590, 1190)	720 (472, 982)	0.13
Monocytes (μL)	310 (240, 425)	310 (248, 422)	300 (230, 405)	0.70
PLT (μL)	187,000 (156,000, 229,000)	185,000 (158,000, 228,000)	201,500 (146,500, 239,750)	0.91
SCr (mg/mL)	0.90 (0.80, 1.10)	0.90 (0.80, 1.00)	1.10 (0.83, 1.30)	0.042
Albumin (g/dL)	37 (32, 40)	38 (33, 41)	34 (30, 36)	0.008
LDH (U/L)	308 (242, 404)	284 (231, 360)	389 (274, 465)	0.021
D-dimer (ng/mL)	1366 (609, 3170)	1180 (513, 1999)	4474 (2516, 4610)	<0.001
Home therapy, *n*(%)				
ACE-inhibitors/ARBs	19/90 (21)	13/68 (19)	6/22 (27)	0.42
Diuretics	17/90 (19)	12/68 (18)	5/22 (23)	0.75
Antiaggregants	14/94 (15)	9/69 (13)	5/25 (20)	0.51
Anticoagulants	4/94 (4.3)	3/69 (4.3)	1/25 (4.0)	>0.99
Statins	12/90 (13)	9/68 (13)	3/22 (14)	>0.99
Outcomes, *n* (%)				
Death	28/100 (28)	8/71 (11)	20/29 (69)	<0.001
Thrombotic event	19/100 (19)	7/71 (9.9)	12/29 (41)	<0.001
Markers, median (IQR)				
sE-selectin (ng/mL)	26.1 (18.1, 35.0)	24.1 (17.0, 30.1)	36.6 (25.8, 47.1)	<0.001

^1^ Median (IQR) or Frequency (%). ^2^ Wilcoxon rank sum test; Pearson’s Chi-squared test. ICU: intensive care unit; CAD, coronary artery disease; ISH, isolated systolic hypertension; CHF, chronic heart failure; AF, atrial fibrillation; PAD, peripheral artery disease; CVA/TIA, cerebral vascular accident/transient ischemic attack; COPD, chronic obstructive pulmonary disease; CCI, Charlson comorbidity index; WBCs, white blood cells; PMNLs, polymorphonuclear leukocytes; PLTs, platelets; SCr, serum creatinine; LDH, lactate dehydrogenase; ARBs, angiotensin receptor blockers.

**Table 2:** sE-selectin as predictor of ICU admission from Simple Logistic Regression.

Analysis			
Characteristic	OR ^1^	95% CI ^1^	*p*-Value
sE-selectin	1.07	1.03, 1.12	<0.001

^1^ OR: odds ratio; CI: confidence interval; ICU: intensive care unit.

**Table 3:** Test performance of sE-selectin for prediction of ICU admission.

Prediction	Optimal Cutoff ^1^ (ng/mL)	Se	Sp	Acc
ICU vs. Non-ICU	32.7	0.61	0.83	0.77

^1^ Optimal cutoff values derived from receiver operating characteristics by Youden’s index and sensitivity (Se), specificity (Sp) and Accuracy (Acc) from the resulting 2 × 2 tables. ICU: intensive care unit.

**Table 4:** Risk factors associated with ICU admission.

Characteristic.	Univariable			Multivariable		
	OR ^1^	95% CI ^1^	*p*-Value	OR ^1^	95% CI ^1^	*p*-Value
Male Sex	4.11	1.37–15.3	0.019			
PMNL > 8000/μL	12.5	3.61–51.6	<0.001			
LDH > 300 U/L	2.64	1.00–7.63	0.058			
sE-selectin > 33 ng/mL	7.39	2.66–21.8	<0.001	13.7	3.25–82.0	0.001
PaO_2_/FiO_2_ ratio						
200–300	7.14	1.67–37.4	0.011	9.08	1.74–62.1	0.013
<200	46.7	11.2–264	<0.001	80.3	14.4–716	<0.001

^1^ OR: odds ratio; CI: confidence interval; ICU: intensive care unit; PMNL: polymorphonuclear leukocytes; LDH: lactate dehydrogenase.

## Data Availability

All data relevant to the study is included in the article and is available from the corresponding author upon request.
